# Teriparatide treatment of osteoporosis in solid organ transplant recipients—a single-center experience

**DOI:** 10.1007/s11657-026-01751-4

**Published:** 2026-09-02

**Authors:** Talia Diker Cohen, Ilana Shraga-Slutzky, Keren Kaminer, Alexander Gorshtein, Idit Dotan, Gloria Tsvetov

**Affiliations:** 1 Institute of Endocrinology, Diabetes and Metabolism, Rabin Medical Center, Beilinson Campus, Petah Tikva, Israel; 2 Gray Faculty of Medicine and Health, Tel Aviv University, Tel Aviv, Israel

**Keywords:** Transplantation, Osteoporosis, Fractures, Teriparatide

## Abstract

***Summary*:**

Solid organ transplant recipients face high fracture risk with limited evidence guiding anabolic therapy. In this real-world cohort, teriparatide was associated with significant improvements in bone mineral density and acceptable safety without adverse graft effects. These findings support selective anabolic treatment in high-risk transplant recipients and highlight the need for prospective studies.

**Purpose:**

Solid organ transplant (SOT) recipients are at high risk for osteoporosis and fragility fractures due to pre-existing organ dysfunction and long-term immunosuppressive therapy, particularly glucocorticoids. However, evidence supporting the use of anabolic osteoporosis therapy in this population remains limited.

**Methods:**

We conducted a retrospective cohort study at a large transplant center including adult kidney, lung, and liver transplant recipients selected for teriparatide treatment based on clinical indications for severe post-transplant osteoporosis for at least 3 months. Bone mineral density (BMD), incident fractures, biochemical parameters, renal function, and adverse events were evaluated during treatment and follow-up.

**Results:**

Thirty-nine SOT recipients (33% men; mean age 61.3 ± 11.6 years) were included, comprising kidney (*n* = 10), lung (*n* = 20), and liver (*n* = 9) transplant recipients. Prior fragility fractures were present in 90% of patients, and 79% had multiple fractures. Median teriparatide treatment duration was 22.3 months. Significant increases in BMD were observed at the lumbar spine (+11 ± 15%, *p* = 0.01), femoral neck (+8 ± 14%, *p* = 0.05), and total hip (+11 ± 16%, *p* = 0.05). Six patients sustained fractures after teriparatide initiation; only two occurred during active therapy, whereas four occurred after treatment discontinuation. Hypercalcemia occurred in one patient and led to treatment cessation. The estimated glomerular filtration rate declined modestly during the first 6 months and stabilized thereafter; a broadly similar pattern was observed in contemporaneous matched transplant controls. No episodes of graft rejection were observed.

**Conclusions:**

In this real-world cohort of SOT recipients with severe osteoporosis, teriparatide therapy was associated with significant improvements in BMD, acceptable safety, and no apparent excess risk to graft function. These findings support the selective use of anabolic therapy in high-risk transplant recipients and highlight the need for prospective studies to define optimal treatment strategies.

**Supplementary Information:**

The online version contains supplementary material available at https://doi.org/10.1007/s11657-026-01751-4.

## Introduction

Solid organ transplant (SOT) recipients are at markedly increased risk for osteoporosis and fragility fractures due to pre-existing organ dysfunction, frequent vitamin D deficiency, and chronic immunosuppressive therapy, most notably glucocorticoid-induced osteoporosis [1, 2]. Guidelines for diagnosis and treatment of osteoporosis in transplant patients are available [1], and several were issued specifically for heart [3], kidney [4], and liver [5] transplant recipients but not for lung transplant recipients. However, data on the efficacy and safety of anti-osteoporotic treatment in this population are limited. While bisphosphonates remain the most commonly used agents for prevention and treatment of osteoporosis in this population, their utility is often limited by renal dysfunction, prompting consideration of alternative therapies [6].

Teriparatide is a recombinant parathyroid hormone (PTH) analogue. It is an effective anabolic treatment for osteoporosis in post-menopausal women [7] and men [8], as well as for glucocorticoid-induced osteoporosis [9]. However, its role in SOT recipients is less defined. There has been only one randomized trial on the use of teriparatide in kidney transplant recipients (6 months, 26 patients) [10] and several small case reports and retrospective studies [11–15]; none has shown data about fractures.

## Purpose

The aim of this study was to describe a large transplant center experience in treatment of post-transplant osteoporosis with teriparatide, including efficacy measures (fractures, bone density) and safety measures (hypercalcemia, hypercalciuria, kidney function, graft rejection).

## Methods

### Study design and population

We conducted a retrospective cohort study using electronic medical records (EMR) at Rabin Medical Center, the largest solid organ transplant center in Israel. The study was approved by the local Helsinki Ethics Committee.

Adult SOT recipients (kidney, lung, liver, or heart) who received teriparatide therapy at any time after transplantation for a minimum duration of 3 months were eligible for inclusion. The study population did not represent an unselected transplant cohort but rather a clinically selected subgroup.

Initiation and duration of teriparatide therapy were not protocol-driven but reflected routine clinical practice and individualized physician decision-making. Treatment was typically initiated in patients with severe osteoporosis, defined by low bone mineral density and/or prior fragility fractures, particularly in those with inadequate response, intolerance, or contraindications to antiresorptive therapy.

In addition, access to teriparatide in Israel is largely regulated through national reimbursement criteria, which preferentially support its use in patients with severe or glucocorticoid-induced osteoporosis.

Patients were followed from initiation of teriparatide therapy until treatment discontinuation and subsequent clinical follow-up, when available.

### Data collection

For each eligible patient, electronic medical records were reviewed for demographic characteristics (age and sex), transplanted organ type, body mass index (BMI), and osteoporosis risk factors, including diabetes mellitus, menopausal status, male hypogonadism, and smoking. Data were also collected on prior anti-osteoporotic therapies, bone mineral density (BMD) assessed by dual-energy X-ray absorptiometry (DXA), and history of fragility fractures before and after teriparatide initiation. Treatment duration was determined from prescription records, along with information on calcium and vitamin D supplementation and immunosuppressive therapy, including glucocorticoid use.

### Bone outcomes

Osteoporosis was defined as a BMD T-score ≤ −2.5 standard deviations in at least one skeletal site (lumbar spine, femoral neck, total hip, or distal radius).

Changes in BMD were assessed using paired DXA measurements obtained at the initiation and at the end of teriparatide therapy, when available. This approach was chosen to ensure that observed changes corresponded to the treatment period. Incident fractures after teriparatide initiation were identified from clinical documentation and imaging reports.

### Laboratory assessments

Biochemical parameters collected included serum albumin-corrected calcium, serum creatinine, estimated glomerular filtration rate (eGFR), serum 25-hydroxyvitamin D (25(OH)D), and 24-h urine calcium excretion when available.

Baseline laboratory values were defined as measurements obtained within 6 months prior to teriparatide initiation. Follow-up laboratory values were collected at predefined time points during therapy, at the end of teriparatide treatment, and at 3–6 months after treatment discontinuation, when available. For each time point, mean stable serum calcium and creatinine values were used for analysis.

Hypercalcemia was defined as an albumin-corrected serum calcium concentration > 10.5 mg/dL.

Estimated GFR (mL/min/1.73 m^2^) was calculated using the Modification of Diet in Renal Disease (MDRD) equation [16].

Renal function was assessed longitudinally using serial eGFR measurements. Changes in eGFR over time within the teriparatide-treated cohort were evaluated descriptively and using within-subject statistical comparisons. Clinically significant renal deterioration was defined as a decline in eGFR to < 30 mL/min/1.73 m^2^ or progression to end-stage kidney disease.

For contextual comparison, eGFR trends were descriptively summarized in a contemporaneous cohort of transplant recipients not treated with teriparatide (“control group”). Controls were matched 1:1 to teriparatide-treated patients by sex, age at transplantation, and transplanted organ. For each teriparatide user, the interval between transplantation and initiation of teriparatide was applied to the contemporaneous matched control to define the index date for follow-up. Serum creatinine levels in controls were collected at the index date, 6 months, and 2 years thereafter. The control group was included solely to provide contextual comparison for longitudinal renal function and was not intended for formal comparative analyses of skeletal outcomes or treatment effects.

### Statistical analysis

Descriptive statistics were used to summarize baseline characteristics and outcomes. Continuous variables are presented as mean ± standard deviation or median (range), as appropriate. Categorical variables are presented as number and percentage.

Changes in continuous variables over time within individuals were analyzed using paired statistical tests or repeated-measures analysis of variance (ANOVA), as appropriate, accounting for within-subject correlations. Between-group comparisons were performed using independent-sample *T* tests for continuous variables and chi-square or Fisher’s exact tests for categorical variables.

All statistical analyses were performed using Microsoft Excel (version 2511). A two-sided *p* value < 0.05 was considered statistically significant.

## Results

### Baseline characteristics

Thirty-nine teriparatide-treated transplant patients were included in the final analysis (10 kidney, 20 lung, 9 liver; although heart transplant recipients were eligible, none met inclusion criteria). Baseline characteristics of the teriparatide-treated cohort and the matched control group (included for renal function context) are summarized in Table 1. The two groups were well matched for age, sex, and transplanted organ, reflecting the predefined matching strategy.

**Table 1:** Baseline characteristics: teriparatide and control group

	Teriparatide (*n* = 39)	Control (*n* = 39)
Age (years), mean ± stdev (median)	61.3 ± 11.6 (63)	61.1 ± 11.2 (63)
Sex, *n* (%)	Male 13 (33%) Female 26 (67%)	Male 13 (33%) Female 26 (67%)
Organ transplanted	Kidney *n* = 10 Lung *n* = 20 Liver *n* = 9 Heart *n* = 0	Kidney *n* = 10 Lung *n* = 20 Liver *n* = 9 Heart *n* = 0
Previous fracture, *n* (%)	35 (90%)	8 (18%)
Multiple fractures, *n* (%)	31 (79%)	5 (13%)
BMD T score ≤ (−2.5), *n* (%)	25/36 (69%)	15/30 (50%)
Previous treatment for osteoporosis, *n* (%)		
Oral bisphosphonates Zoledronic acid Denosumab Romosozumab None	23 (59%) 5 (13%) 2 (5%) 0 (0%) 14 (36%)	14 (36%) 1 (3%) 4 (10%)^a^ 1 (3%) 20 (51%)
Anti-rejection drugs, *n* (%)		
Steroids CNI mTOR inhibitors	39 (100%) 38 (97%) 2 (5%)	32 (82%) 36 (92%) 2 (5%)
Diabetes mellitus, *n* (%)	15 (38%)	22 (56%)
Nephrolithiasis, *n* (%)	1 (3%)	2 (5%)
Corrected calcium (mg/dL) Vitamin D (nmol/L)	9.1 ± 1.1 66.8 ± 23.2	9.5 ± 0.6 57.1 ± 25.5

*BMD* bone mineral density, *CNI* calcineurin inhibitors, *mTOR* mechanistic target of rapamycin

^a^One patient had been treated with oral bisphosphonates before denosumab therapy

As expected, the teriparatide-treated group differed substantially from controls in clinical characteristics, consistent with treatment selection based on disease severity. Patients receiving teriparatide had a markedly higher prevalence of prior fragility fractures (90% vs 18%) and multiple fractures (79% vs 13%), as well as a higher proportion of osteoporotic-range BMD (69% vs 50%). Prior use of anti-osteoporotic therapy was also more common in the teriparatide group.

These differences confirm that the treated cohort represents a selected high-risk population with more advanced skeletal disease compared with untreated transplant recipients.

### Teriparatide exposure

Clinical and biochemical characteristics of the teriparatide-treated cohort at treatment initiation and at the end of therapy are summarized in Table 2. Teriparatide therapy was initiated at variable time points after transplantation, with a median interval of 36 months (IQR 15–102, mean 73 ± 94 months). Treatment duration was also variable, with a median of 22.3 months (range 3.1–25.3), reflecting real-world clinical practice rather than a predefined treatment protocol.

**Table 2:** Clinical and biochemical characteristics of patients treated with teriparatide

Teriparatide group (*n* = 39)	At start of treatment	End of treatment
Osteoporosis risk factors, *n* (%)		
Diabetes mellitus Menopause Male hypogonadism Smoking BMI, kg/m^2^	15 (38%) 22 (85%) 5 (38%) 1 (3%) 25.0 ± 4.2	
Hypoparathyroidism, *n* (%)	3 (8%)	
Calcium supplement, *n* (%)	31 (79%)	
Vitamin D supplement, *n* (%)	37 (95%)	
Fragility fractures, *n* (%)	Prior fractures (before teriparatide therapy) 35 (90%)	Incident fractures (after the initiation of teriparatide) 6 (17%)
BMD T score ≤ (−2.5), *n* (%)		
Lumbar spine Femoral neck Total hip	17/33 (52%) 22/36 (61%) 12/19 (63%)	6/20 (30%) 9/22 (41%) 8/18 (44%)
Corrected calcium (mg/dL) Phosphorus (mg/dL) Vitamin D (nmol/L) Uric acid (mg/dL) Urine calcium (mg/day)	9.1 ± 1.1 3.8 ± 0.6 66.8 ± 23.2 5.6 ± 1.5 124.0 ± 91.4	9.3 ± 1.2 3.3 ± 0.7 76.3 ± 24.9 6.5 ± 1.3 98.6 ± 55.4

*BMD* bone mineral density, *ND* not available/not systematically collected

Teriparatide was used as first-line osteoporosis therapy in 36% of patients, while the remainder had received prior antiresorptive treatment, most commonly oral bisphosphonates (59%) or zoledronic acid (13%). The average follow-up since the start of teriparatide was 6.8 years. Concomitant calcium and vitamin D supplementation was prescribed in 79% and 95% of patients, respectively. All patients were receiving glucocorticoids at teriparatide initiation (mean daily dose of 7.0 ± 3.6 mg).

### Skeletal outcomes

Six patients sustained fractures after initiation of teriparatide therapy; detailed characteristics are shown in Online Resource 1. Importantly, only two fractures occurred during active teriparatide treatment, whereas four fractures occurred after treatment discontinuation, with variable latency ranging from 4 months to more than 10 years after cessation.

**Fig. 1:**
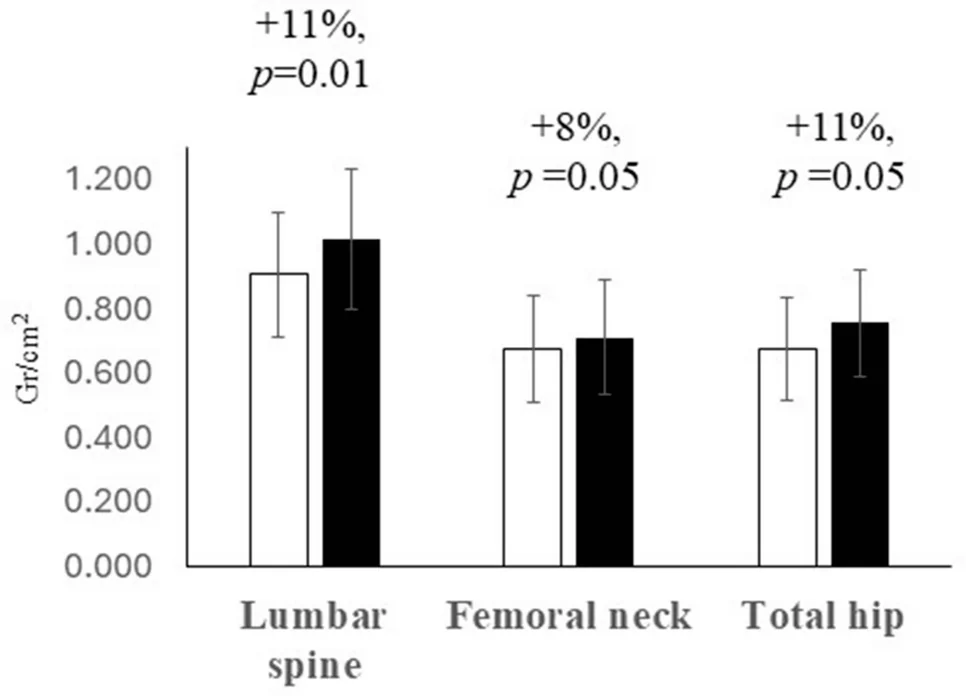
Changes in bone mineral density during teriparatide therapy in solid organ transplant recipients. Bone mineral density (g/cm^2^) at the lumbar spine, femoral neck, and total hip measured by dual-energy X-ray absorptiometry in patients with paired measurements at initiation (white bars) and completion (black bars) of teriparatide therapy. Data are shown for the subset of patients with available paired measurements (lumbar spine *n* = 18, femoral neck *n* = 17, total hip *n* = 11). Significant increases were observed at all skeletal sites

Among patients with appropriately paired DXA measurements at treatment initiation and completion, BMD data were available for 18 (46%), 17 (44%), and 11 (28%) patients at the lumbar spine, femoral neck, and total hip, respectively. In this subset, significant increases in BMD were observed at all evaluated skeletal sites. Lumbar spine BMD increased by 11 ± 15% (*p* = 0.01), femoral neck BMD by 8 ± 14% (*p* = 0.05), and total hip BMD by 11 ± 16% (*p* = 0.05) (Fig. 1).

### Biochemical and renal safety

Changes in biochemical parameters during teriparatide therapy are summarized in Table 2. Hypercalcemia occurred in one patient (corrected calcium increased from 9.6 to 10.9 mg/dL during the second year of therapy), leading to teriparatide discontinuation. No cases of hypercalciuria were detected.

**Fig. 2:**
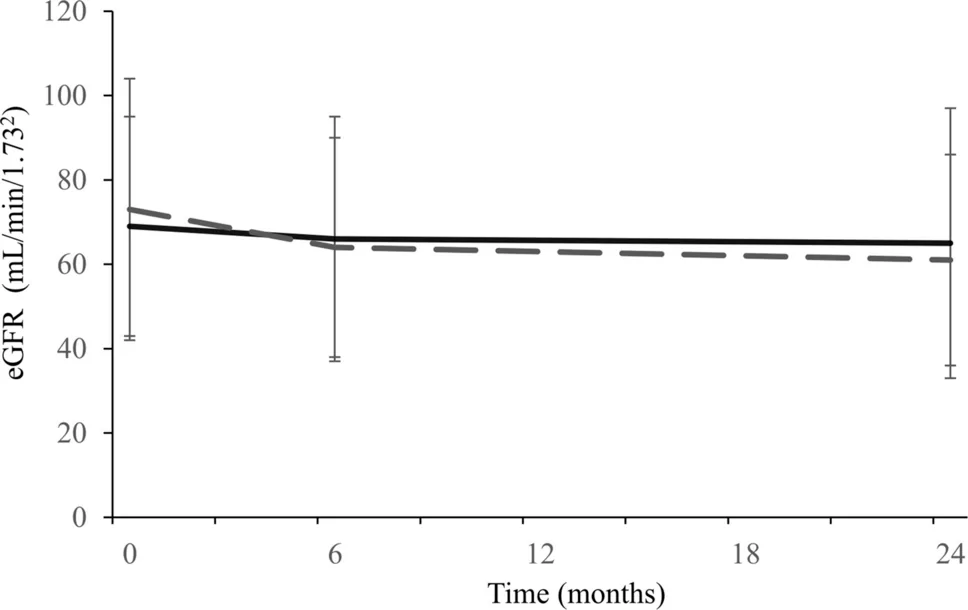
Longitudinal kidney function in transplant recipients treated with teriparatide compared with untreated controls. Estimated glomerular filtration rate (eGFR) over time in teriparatide-treated transplant recipients (dashed line) and untreated controls (solid line), showing an early modest decline followed by stabilization over time in both groups

Mean eGFR declined modestly during the first 6 months of teriparatide therapy (from 73 ± 31 to 64 ± 26 mL/min/1.73 m^2^) and remained stable thereafter, including after treatment discontinuation (62 ± 27 mL/min/1.73 m^2^ at 3–6 months post-therapy) (Table 3, Fig. 2). Repeated-measures ANOVA demonstrated a significant change in eGFR over time, driven by an early decline from baseline followed by stabilization during continued treatment and post-treatment follow-up (*p* < 0.001). Importantly, a similar magnitude of eGFR decline was observed in a contemporaneous matched control group of transplant recipients not treated with teriparatide (Fig. 2).

**Table 3:** Kidney function over time

	Baseline	6 months	2 years	3–6 months after end of teriparatide
Creatinine				
Control Teriparatide Significance^a^	1.0 ± 0.3 1.0 ± 0.3 *p* = 0.783	1.1 ± 0.5 1.1 ± 0.4 *p* = 0.734	1.2 ± 0.6 1.2 ± 0.5 *p* = 0.528	1.1 ± 0.4
Estimated GFR				
Control Teriparatide Significance^a^	69 ± 26 73 ± 31 *p* = 0.633	66 ± 29^b^ 64 ± 26^b^ *p* = 0.626	65 ± 32 61 ± 25 *p* = 0.505	62 ± 27
Estimated GFR—change from Baseline				
Control Teriparatide Significance^a^		−3 ± 17 −9 ± 18 *p* = 0.115	−4 ± 17 −13 ± 21 *p* = 0.056	−12 ± 20

*GFR* glomerular filtration rate

^a^Significance—comparison between control and teriparatide at the same time point

^b^*p* < 0.05 for result at 6 months compared to result at baseline

Five patients experienced a decline in eGFR to < 30 mL/min/1.73 m^2^ during follow-up; all had baseline eGFR values between 30 and 39 mL/min/1.73 m^2^. Only one patient discontinued teriparatide after 8 months of treatment. No patient progressed to end-stage kidney disease or required renal replacement therapy.

### Other adverse events

Five patients reported non-renal adverse events, including myalgia (*n* = 2), nausea (*n* = 2), and dizziness (*n* = 1). Twenty-one patients were hospitalized during teriparatide therapy, most commonly due to infections (*n* = 9). No episodes of graft rejection occurred during treatment.

Nine patients died during study follow-up, including seven deaths within the first 2 years of teriparatide initiation; deaths were attributed to underlying comorbidities rather than treatment-related causes.

### Sex-based analyses

Sex-stratified analyses are presented in Table 4. Age, fracture history, treatment duration, and baseline BMD did not differ between women and men. Diabetes mellitus was significantly more prevalent among male recipients (70% vs 23%, *p* = 0.01).

**Table 4:** Comparison between male and female subjects

	Female (*n* = 26)	Male (*n* = 13)	
Age (years), mean ± stdev	62.3 ± 11.6	59.2 ± 11.8	*p* = 0.44
Organ transplanted	Kidney *n* = 8 Lung *n* = 12 Liver *n* = 6 Heart *n* = 0	Kidney *n* = 2 Lung *n* = 8 Liver *n* = 3 Heart *n* = 0	*p* = 0.51
Duration of teriparatide therapy (months), median (min, max)	21.5 (3.1, 25.3)	23.8 (6.1, 24.7)	*p* = 0.35
Previous fracture, *n* (%)	23 (88%)	12 (92%)	*p* = 0.71
Multiple fractures, *n* (%)	19 (73%)	12 (92%)	*p* = 0.16
Previous treatment for osteoporosis, *n* (%)			
MHT/SERM/testosterone Oral bisphosphonates Zoledronic acid Denosumab None	14 (54%) 15 (58%) 5 (19%) 2 (8%) 9 (35%)	0 (0%) 8 (62%) 0 (0%) 0 (0%) 5 (38%)	N/A *p* = 0.82 N/A N/A *p* = 0.81
Calcium supplement, *n* (%)	20 (77%)	10 (77%)	*p* = 1.00
Vitamin D supplement, *n* (%)	25 (96%)	25 (92%)	*p* = 0.35
Anti-rejection drugs, *n* (%) Steroids CNI mTOR inhibitors	26 (100%) 25 (96%) 2 (8%)	13 (100%) 13 (100%) 0 (0%)	N/A N/A N/A
Risk factors for decreased bone density, *n* (%)			
Diabetes mellitus Menopause Male hypogonadism Smoking BMI, kg/m^2^, mean ± stdev	6 (23%) 22 (85%) N/A 1 (4%) 25.0 ± 4.8	9 (70%) N/A 5 (38%) 0 (0%) 24.6 ± 3.5	*p* = 0.01 N/A N/A N/A *p* = 0.77
Nephrolithiasis, *n* (%)	0 (0%)	1 (8%)	N/A
Hypoparathyroidism, *n* (%)	3 (12%)	0 (0%)	N/A
BMD T score ≤ (−2.5), *n* (%) Lumbar spine Femoral neck Total hip	12 (46%) 16 (62%) 10 (38%)	5 (38%) 5 (38%) 2 (15%)	*p* = 0.21 *p* = 0.17 *p* = 0.14
Baseline labs, mean ± stdev			
Corrected calcium (mg/dL) Phosphorus (mg/dL) Creatinine (mg/dL) eGFR (mL/min/1.73^2^) Vitamin D (nmol/L) Urine calcium (mg/day)	9.2 ± 0.6 3.9 ± 0.7 1.0 ± 0.3 65 ± 23 63 ± 17 90 ± 54	9.2 ± 0.5 3.5 ± 0.4 1.0 ± 0.3 89 ± 39 75 ± 30 173 ± 119	*p* = 0.91 *p* = 0.04 *p* = 0.95 *p* = 0.06 *p* = 0.16 *p* = 0.09
eGFR on teriparatide (at 6 months of therapy)	59 ± 24	74 ± 27	*p* = 0.10
eGFR at end of teriparatide	54 ± 22	74 ± 27	*p* = 0.03
eGFR 3–6 months after stop of teriparatide	58 ± 27	75 ± 26	*p* = 0.10
Change in eGFR on teriparatide^a^	−6	−14	*p* = 0.25
Change in eGFR after stop of teriparatide^a^	−7	−14	*p* = 0.53

*MHT* menopausal hormone therapy, *SERM* selective estrogen receptor modulator, *CNI* calcineurin inhibitors, *mTOR* mechanistic target of rapamycin, *BMI* body mass index, *BMD* bone mineral density, *eGFR* estimated glomerular filtration rate

^a^Compared to baseline eGFR

Baseline eGFR was higher in men, as well as at the end of teriparatide therapy. However, the magnitude of eGFR change during and after teriparatide treatment did not differ significantly between sexes.

## Discussion

In this single-center retrospective cohort, we report one of the largest and most heterogeneous real-world cohorts of SOT recipients with osteoporosis treated with teriparatide. Our findings demonstrate that teriparatide treatment was associated with clinically meaningful improvements in bone mineral density at the lumbar spine and hip in this selected high-risk cohort, accompanied by a low incidence of treatment-limiting adverse effects and no apparent increase in graft-related complications. These findings support the potential role of anabolic therapy in carefully selected transplant recipients with severe osteoporosis.

Importantly, the comparison with untreated transplant recipients highlights the strong selection inherent to teriparatide use in clinical practice. Despite matching for demographic and transplant-related variables, treated patients had substantially more severe skeletal disease at baseline, including a markedly higher burden of prior fractures and lower bone density (Table 1). This reflects appropriate clinical decision-making and reimbursement constraints but limits direct comparison between groups and precludes causal inference regarding treatment effects.

Although teriparatide is an established first-line therapy for glucocorticoid-induced osteoporosis, its effectiveness in transplant recipients remains uncertain. Concerns have been raised regarding potential skeletal resistance to parathyroid hormone after transplantation, attributed to adynamic bone disease and the cumulative effects of long-term immunosuppressive therapy. Both experimental and clinical studies have suggested impaired osteoblastic responsiveness to PTH signaling in chronic kidney disease and post-transplant states [17, 18], thereby questioning the anabolic efficacy of teriparatide in this population. However, evidence directly addressing these concerns in transplant recipients remains limited.

Notably, most published experience with teriparatide in transplant recipients relates to its use for refractory hypocalcemia due to hypoparathyroidism [19–22], particularly after parathyroidectomy, rather than for osteoporosis treatment. These reports primarily focus on biochemical correction, with limited assessment of skeletal outcomes. Only a small number of case reports, small series, or retrospective studies have evaluated the bone effects of teriparatide for osteoporosis, describing modest increases in bone mineral density in lung [11, 14] and kidney [13, 15] transplant recipients and histological evidence of increased bone formation in a heart transplant patient with low-turnover bone disease [12]. Overall, data on the skeletal efficacy of teriparatide in transplant populations remain sparse and largely anecdotal.

To date, there is only one randomized controlled trial on teriparatide use in transplant recipients, conducted by Cejka et al. [10]. The trial evaluated teriparatide initiated early after kidney transplantation for 6 months and did not demonstrate improvements in bone mineral density or histomorphometric parameters. Importantly, therapy in that study was started during a period characterized by intense immunosuppression, high-dose glucocorticoid exposure, and markedly suppressed bone turnover, and treatment duration was relatively short. These factors likely limited the ability to detect meaningful structural skeletal responses. Notably, despite the absence of BMD improvement, bone loss was prevented in the teriparatide group compared with placebo, suggesting a possible protective effect during the early post-transplant period.

In contrast, patients in our cohort initiated teriparatide later after transplantation (similar to other retrospective studies [13–15], see Table 5) and were treated for a median duration exceeding 22 months, reflecting real-world clinical practice in which anabolic therapy is typically considered after stabilization of graft function and immunosuppressive regimens. This later timing, combined with longer exposure, may explain the substantial BMD gains observed in our study. The magnitude of improvement, approximately 11% at the lumbar spine and 8–11% at the hip, is comparable to responses reported in non-transplant populations and exceeds those typically achieved with antiresorptive therapy in transplant recipients. These changes were observed in analyses restricted to patients with paired measurements at treatment initiation and completion and were accompanied by a reduction in the proportion of patients with osteoporotic-range BMD across skeletal sites (Table 2).

**Table 5:** Teriparatide use in solid organ transplant recipients: comparison of published evidence

Study	Age mean (yr)	Sex (male)	Population	Design	N (TP)	Mean time after transplant (months)	Duration (median, months)	BMD outcome	Fractures	Kidney safety
Current study	61 ± 12	33%	Kidney, lung, liver, heart	Retrospective	39	73 ± 94 (median 36 mo)	22.3 (3.1–25.3)	↑ LS, FN, TH	2 during active therapy; 4 after treatment discontinuation	Early ↓ then stable eGFR
Cejka (2008)	51 ± 9	75%	Kidney	RCT (vs placebo)	12:12	≥ 1	6 mo	Stable BMD vs ↓placebo	NR	Stable
Qiu (2021)	47 ± 7	29%	Kidney	Retrospective	68	61 ± 12	12 mo	↑ LS, FN, TH ↑ vs alendronate	1 (colles):5 (3 vert + 2nonvert)	Stable
Vetrano (2025)	64 ± 8	56%	Kidney	Retrospective, single center	18	67 ± 9	12 mo (all pts) to 24 mo (9 pts)	↑ LS, TH	1 (AE)	Stable
January (2025)	60 ± 2	39%	Lung	Comparative cohort (vs alendronate, denosumab)	44	35 ± 15	15.6 mo (6.0–25.2)	↑ LS, hip (vs alendronate)	18% (similar to other groups)	Stable

*TP* teriparatide, *RCT* randomized control study, *BMD* bone mineral density, *LS* lumbar spine, *FN* femoral neck, *TH* total hip

Our findings are consistent with results from recent observational studies in kidney transplant recipients. Qiu et al. demonstrated greater increases in lumbar spine and hip BMD with teriparatide compared with alendronate over 12 months [13], while Vetrano et al. reported significant BMD gains in patients with low bone turnover disease treated with teriparatide in a multicenter Italian experience [15]. Importantly, unlike previous studies restricted to kidney transplantation, our cohort included kidney, lung, and liver transplant recipients, thereby extending the potential applicability of anabolic therapy across multiple transplant populations.

Fracture outcomes in transplant cohorts are challenging to interpret due to severe baseline skeletal disease and limited event numbers. In our study, although fractures occurred in a minority of patients during teriparatide exposure, these events were largely observed in individuals with extremely high baseline fracture burden and advanced osteoporosis. Given the retrospective design, lack of an untreated comparator, and limited fracture event numbers, our study was not powered to detect a fracture-reduction signal. Nevertheless, the stabilization of fracture incidence in a cohort with severe baseline skeletal fragility is clinically reassuring and consistent with fracture trends reported in other transplant series [14].

A key concern limiting the use of osteoporotic therapy in transplant recipients is renal safety. In our cohort, eGFR declined modestly during the first 6 months of teriparatide therapy but subsequently stabilized, including after treatment discontinuation. Importantly, a broadly similar pattern of kidney function change was observed in contemporaneous matched transplant controls not treated with teriparatide. Because the study was not designed or powered to formally compare renal outcomes between groups, and because treated patients represented a clinically selected high-risk population, definitive conclusions regarding the renal effects of teriparatide cannot be drawn. However, no clear signal of accelerated kidney function decline was observed. No patient progressed to end-stage kidney disease or required dialysis during teriparatide treatment or follow-up.

Metabolic adverse events were infrequent. Hypercalcemia occurred in only one patient and resolved with treatment discontinuation. This is consistent with previous reports in transplant recipients [19]. Despite widespread concomitant calcium and vitamin D supplementation, no cases of clinically significant hypercalciuria were observed. These findings are consistent with prior reports and support the metabolic safety of teriparatide in transplant populations when appropriately monitored.

Adverse events were generally mild and consistent with the known safety profile of teriparatide, including musculoskeletal pain, nausea, and dizziness. Treatment discontinuation due to adverse effects was uncommon, reinforcing the tolerability of teriparatide even with prolonged exposure.

Taken together, our findings suggest that teriparatide represents a viable therapeutic option for selected solid organ transplant recipients with severe osteoporosis. While not suitable for universal use, teriparatide may fill a critical treatment gap in high-risk transplant populations where antiresorptive therapy is ineffective or contraindicated.

This study has several strengths and limitations. The retrospective design, absence of a homogeneous control group, and heterogeneity of transplant types limit causal inference. In addition, treatment allocation was not protocol-driven but based on clinical judgment and influenced by national reimbursement policies, resulting in a selected high-risk population enriched for patients with severe or glucocorticoid-induced osteoporosis and introducing potential indication bias. Variability in the timing of treatment initiation and duration further reflects real-world practice but introduces additional heterogeneity. Although a matched contemporaneous control group was included, it was designed solely for contextual comparison of renal function and not for comparative effectiveness analyses, and substantial baseline differences in skeletal risk remained. BMD analyses were restricted to patients with paired measurements aligned with treatment initiation and completion, which reduced sample size but improved internal validity by ensuring that observed changes reflected the treatment period. Bone turnover markers and histomorphometric data were not systematically available, precluding mechanistic insights into anabolic responsiveness. Residual confounding by disease severity and survivor bias cannot be excluded. Nonetheless, the relatively large cohort, extended follow-up, and inclusion of clinically relevant skeletal and safety outcomes distinguish this study from prior reports.

In conclusion, in this large real-world cohort of solid organ transplant recipients, teriparatide therapy was associated with clinically meaningful improvements in bone mineral density, acceptable metabolic and renal safety, and no apparent excess risk to graft function. These findings support the selective use of anabolic therapy in transplant recipients with severe osteoporosis and highlight the need for prospective studies to define optimal patient selection, treatment timing, and sequencing strategies in this complex population.

## Supplementary Information

Below is the link to the electronic supplementary material.

## Data Availability

The data that support the findings of this study are available on request from the corresponding author. The data are not publicly available due to privacy or ethical restrictions.

## References

[1] Anastasilakis AD, Tsourdi E, Makras P, Polyzos SA, Meier C, McCloskey EV, Pepe J, Zillikens MC (2019) Bone disease following solid organ transplantation: a narrative review and recommendations for management from The European Calcified Tissue Society. Bone 127:401–418 31299385 10.1016/j.bone.2019.07.006

[2] Buckley L, Humphrey MB (2018) Glucocorticoid-induced osteoporosis. N Engl J Med 379:2547–2556 30586507 10.1056/NEJMcp1800214

[3] Costanzo MR, Dipchand A, Starling R et al (2010) The International Society of Heart and Lung Transplantation guidelines for the care of heart transplant recipients. J Heart Lung Transplant 29:914–956 20643330 10.1016/j.healun.2010.05.034

[4] Improving Global Outcomes (KDIGO) Transplant Work Group (2009) KDIGO clinical practice guideline for the care of kidney transplant recipients. Am J Transplant 9(Suppl 3):S1–S157 10.1111/j.1600-6143.2009.02834.x 19845597

[5] Lucey MR, Terrault N, Ojo L, Hay JE, Neuberger J, Blumberg E, Teperman LW (2013) Long-term management of the successful adult liver transplant: 2012 practice guideline by the American Association for the Study of Liver Diseases and the American Society of Transplantation. Liver Transpl 19:3–26 23281277 10.1002/lt.23566

[6] Palmer SC, Chung EY, McGregor DO, Bachmann F, Strippoli GF (2019) Interventions for preventing bone disease in kidney transplant recipients. Cochrane Database Syst Rev 10:Cd005015 31637698 10.1002/14651858.CD005015.pub4 PMC6803293

[7] Neer RM, Arnaud CD, Zanchetta JR et al (2001) Effect of parathyroid hormone (1–34) on fractures and bone mineral density in postmenopausal women with osteoporosis. N Engl J Med 344:1434–1441 11346808 10.1056/NEJM200105103441904

[8] Finkelstein JS, Hayes A, Hunzelman JL, Wyland JJ, Lee H, Neer RM (2003) The effects of parathyroid hormone, alendronate, or both in men with osteoporosis. N Engl J Med 349:1216–1226 14500805 10.1056/NEJMoa035725

[9] Saag KG, Shane E, Boonen S, Marín F, Donley DW, Taylor KA, Dalsky GP, Marcus R (2007) Teriparatide or alendronate in glucocorticoid-induced osteoporosis. N Engl J Med 357:2028–2039 18003959 10.1056/NEJMoa071408

[10] Cejka D, Benesch T, Krestan C, Roschger P, Klaushofer K, Pietschmann P, Haas M (2008) Effect of teriparatide on early bone loss after kidney transplantation. Am J Transplant 8:1864–1870 18786230 10.1111/j.1600-6143.2008.02327.x

[11] Raven LM, Goodall L, Center JR, Muir CA (2024) Teriparatide as treatment for severe osteoporosis in lung transplant recipients. JCEM Case Rep 2:luae026 38495394 10.1210/jcemcr/luae026 PMC10943497

[12] Fahrleitner-Pammer A, Wagner D, Krisper P, Amrein K, Dimai H (2017) Teriparatide treatment in a heart transplant patient with a chronic kidney disease and a low-turnover bone disease: a case report. Osteoporos Int 28:1149–1152 27988794 10.1007/s00198-016-3858-2 PMC5406430

[13] Qiu Z, Lin C, Zhang Y, Lin C, Deng J, Wu M (2021) Analysis of the efficacy and safety of teriparatide and alendronate in the treatment of osteoporosis after renal transplantation. Iran J Kidney Dis 15:451–456 34930857 10.52547/ijkd.6475

[14] January SE, Fester KA, Escamilla JE, Cano M (2025) Comparative safety and efficacy of bisphosphonates, denosumab, and parathyroid hormone analogs for osteoporosis following lung transplantation. Pharmacotherapy 45:495–503 40605738 10.1002/phar.70040

[15] Vetrano D, Aguanno F, Passaseo A, Barbuto S, Tondolo F, Catalano V, Zavatta G, Pagotto U, La Manna G, Cianciolo G (2025) Efficacy and safety of teriparatide in kidney transplant recipients with osteoporosis and low bone turnover: a real-world experience. Int Urol Nephrol 57:1965–1975 39871033 10.1007/s11255-025-04383-8 PMC12049390

[16] Levey AS, Coresh J, Greene T, Stevens LA, Zhang YL, Hendriksen S, Kusek JW, Van Lente F (2006) Using standardized serum creatinine values in the modification of diet in renal disease study equation for estimating glomerular filtration rate. Ann Intern Med 145:247–254 16908915 10.7326/0003-4819-145-4-200608150-00004

[17] Iwasaki-Ishizuka Y, Yamato H, Nii-Kono T, Kurokawa K, Fukagawa M (2005) Downregulation of parathyroid hormone receptor gene expression and osteoblastic dysfunction associated with skeletal resistance to parathyroid hormone in a rat model of renal failure with low turnover bone. Nephrol Dial Transplant 20:1904–1911 15985520 10.1093/ndt/gfh876

[18] Velasquez-Forero F, Mondragón A, Herrero B, Peña JC (1996) Adynamic bone lesion in renal transplant recipients with normal renal function. Nephrol Dial Transplant 11(Suppl 3):58–64 8840319 10.1093/ndt/11.supp3.58

[19] Hod T, Riella LV, Chandraker A (2015) Recombinant PTH therapy for severe hypoparathyroidism after kidney transplantation in pre-transplant parathyroidectomized patients: review of the literature and a case report. Clin Transplant 29:951–957 26331695 10.1111/ctr.12622

[20] León L, Rial MC, Curcio D, Casadei D (2013) Teriparatide in kidney transplant patients with severe hypoparathyroidism. Nefrologia 33:601–602 23897194 10.3265/Nefrologia.pre2013.Apr.11933

[21] Mahajan A, Narayanan M, Jaffers G, Concepcion L (2009) Hypoparathyroidism associated with severe mineral bone disease postrenal transplantation, treated successfully with recombinant PTH. Hemodial Int 13:547–550 19493022 10.1111/j.1542-4758.2009.00380.x

[22] Nogueira EL, Costa AC, Santana A, Guerra JO, Silva S, Mil-Homens C, Costa AG (2011) Teriparatide efficacy in the treatment of severe hypocalcemia after kidney transplantation in parathyroidectomized patients: a series of five case reports. Transplantation 92:316–320 21694663 10.1097/TP.0b013e3182247b98

