# Supplementary material for: Teriparatide treatment of osteoporosis in solid organ transplant recipients—a single-center experience
Source: Arch Osteoporos. 2026 Sep 2;21(1):136. doi: 10.1007/s11657-026-01751-4 (PMC13538171; doi:10.1007/s11657-026-01751-4)
Supplement: Supplementary file 1 [file 11657_2026_1751_MOESM1_ESM.pdf]

Online Resource 1. Characteristics of cases with fractures after the start of teriparatide treatment

	Case 1	Case 2	Case 3	Case 4	Case 5	Case 6
Gender	Female	Male	Female	Female	Female	Female
Age (years)	69	72	62	71	59	60
Organ transplanted	Liver	Kidney	Lung	Lung	Liver	Liver
Duration of teriparatide treatment (months)	22	15	24	24	24	24
Previous fracture	N	Y	Y	Y	Y	N
Previous multiple fractures	N	Y	Y	N	Y	N
Prednisone dose (mg/day)	10	5	7.5	5	0	2.5
Fracture site after start of teriparatide	Vertebrae	Femoral neck	Ankle	Vertebrae	Vertebrae	Humerus+2 vertebrae
Timing of fractures post-teriparatide	4 months after stop of teriparatide	7 months after stop of teriparatide	12 months after start of teriparatide	4 months after stop of teriparatide	129 months after stop of teriparatide	4 months after start of teriparatide
